# Impact of Altered Body Composition on Clinical and Oncological Outcomes in Intrahepatic Cholangiocarcinoma

**DOI:** 10.3390/jcm12247747

**Published:** 2023-12-18

**Authors:** Guanwu Wang, Carlos C. Otto, Lara R. Heij, Tarick M. Al-Masri, Edgar Dahl, Daniel Heise, Steven W. M. Olde Damink, Tom Luedde, Sven A. Lang, Tom F. Ulmer, Ulf P. Neumann, Jan Bednarsch

**Affiliations:** 1 Department of Surgery and Transplantation, University Hospital RWTH Aachen, 52074 Aachen, Germany; gwang@ukaachen.de (G.W.); carlosconstantin.otto@uk-essen.de (C.C.O.); lheij@ukaachen.de (L.R.H.); talmasri@ukaachen.de (T.M.A.-M.); daniel.heise@uk-essen.de (D.H.); svenarke.lang@uk-essen.de (S.A.L.); fulmer@ukaachen.de (T.F.U.); ulf.neumann@uk-essen.de (U.P.N.); 2 Department of Surgery and Transplantation, University Hospital Essen, 45147 Essen, Germany; 3 University of Applied Science Aachen, 52066 Aachen, Germany; 4 Institute of Pathology, University Hospital RWTH Aachen, 52074 Aachen, Germany; edahl@ukaachen.de; 5 Department of Surgery, Maastricht University Medical Centre (MUMC), 6229 HX Maastricht, The Netherlands; steven.oldedamink@mumc.nl; 6 Department of Gastroenterology, Hepatology and Infectious Diseases, Heinrich Heine University Duesseldorf, 40225 Duesseldorf, Germany; tom.luedde@med.uni-duesseldorf.de

**Keywords:** intrahepatic cholangiocarcinoma, body composition, postoperative complications, oncological outcome

## Abstract

Intrahepatic cholangiocarcinoma is a common primary liver tumor with limited treatment options and poor prognosis. Changes in body composition (BC) have been shown to affect the prognosis of various types of tumors. Therefore, our study aimed to investigate the correlation between BC and clinical and oncological outcomes in patients with iCCA. All patients with iCCA who had surgery from 2010 to 2022 at our institution were included. We used CT scans and 3D Slicer software to assess BC and conducted logistic regressions as well as Cox regressions and Kaplan–Meier analyses to investigate associations between BC and clinical variables with focus on postoperative complications and oncological outcomes. BC was frequently altered in iCCA (*n* = 162), with 53.1% of the patients showing obesity, 63.2% sarcopenia, 52.8% myosteatosis, 10.1% visceral obesity, and 15.3% sarcopenic obesity. The multivariate analysis showed no meaningful association between BC and perioperative complications. Myosteatosis was associated with reduced overall survival (OS) in iCCA patients (myosteatosis vs. non-myosteatosis, 7 vs. 18 months, *p* = 0.016 log rank). Further, the subgroup analysis revealed a notable effect in the subset of R0-resected patients (myosteatosis vs. non-myosteatosis, 18 vs. 32 months, *p* = 0.025) and patients with nodal metastases (myosteatosis vs. non-myosteatosis, 7 vs. 18 months, *p* = 0.016). While altered BC is not associated with perioperative outcomes in iCCA, myosteatosis emerges as a prognostic factor for reduced OS in the overall and sub-populations of resected patients.

## 1. Introduction

Intrahepatic cholangiocarcinoma (iCCA) is the second most common primary liver cancer and constitutes approximately 20% of liver tumors and 3% of all gastrointestinal malignancies [1]. Surgical resection alone remains the sole curative option for patients diagnosed with localized iCCA [2,3]. Nevertheless, due to its asymptomatic nature during the initial stages, most patients present in advanced stages, making them ineligible for curative-intent surgery [2]. The prognosis for iCCA patients remains dismal, as a 5-year survival rate of around 9% has been reported, with cancer-related deaths mainly due to early metastatic recurrence and the limited effectiveness of systemic therapy [4]. In consideration of these circumstances, there arises a compelling need to identify prognostic markers of heightened sensitivity, with the overall goal of refining the management approaches for patients diagnosed with iCCA. Body composition (BC) has emerged as a valuable predictor of clinical outcomes and prognosis in cancer patients [5]. The European Society of Clinical Nutrition and Metabolism advocates the assessment of BC as a critical step in the evaluation of the nutritional status of individuals with cancer [6]. In particular, the assessment of skeletal muscle, adipose tissue, and bone is important as an integral part of nutritional assessment, especially in the context of cancer cachexia. Changes in BC, encompassing parameters such as body mass index (BMI), sarcopenia, myosteatosis, visceral obesity, and sarcopenic obesity, assume the role of prognostic indicators across diverse clinical contexts, including in the context of hepatocellular carcinoma (HCC), gastric cancer, and breast cancer [7,8,9]. The primary objective of this study was to evaluate the effect of preoperative BC on the perioperative and oncologic outcomes of patients with iCCA.

## 2. Materials and Methods

### 2.1. Study Population

This study is a retrospective, single-center cohort investigation involving patients who underwent iCCA surgery at RWTH Aachen University Hospital between May 2009 and December 2022. The eligible patients were required to have computed tomography (CT) scans conducted up to three months before their surgery. A total of 162 patients diagnosed with iCCA were enrolled in this study. In this study, we collected clinicopathological and survival data from a prospective institutional database. The research adhered to the current version of the Declaration of Helsinki and followed the guidelines of good clinical practice (International Conference on Harmonization, Good Clinical Practice). Further, this study received approval from the Ethics Committee of RWTH Aachen University Hospital (EK 23-269).

### 2.2. Body Composition Measurement

**Figure 1:**
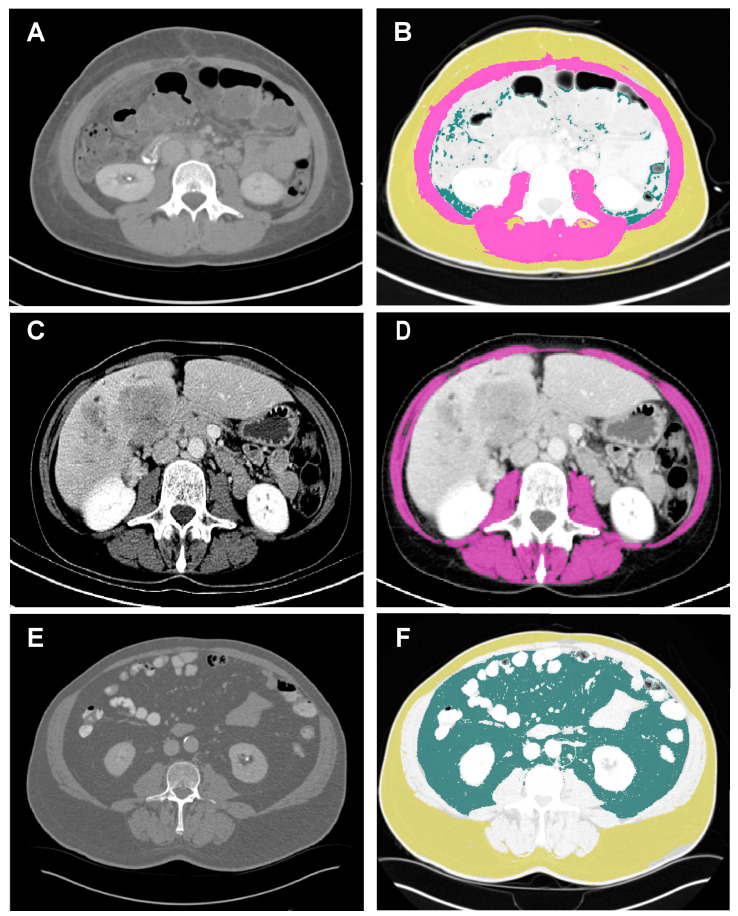
Example of a body composition measurement. Examples of BC analysis. Images displaying a C scan orientated at the third lumbar vertebra in patients with iCCA. The analyzed regions were defined using the following attenuation values: skeletal muscle region (purple), −29–150 HU; subcutaneous fat region (yellow), −190~−30 HU; visceral fat region (dark green), −150~−50 HU. (**A**,**B**) Patient with normal BC. (**C**,**D**) Patient with sarcopenia and myosteatosis. (**E**,**F**) Patient with visceral obesity. BC, body composition. HU, Hounsfield unit; iCCA, intrahepatic cholangiocarcinoma.

Comprehensive CT imaging for the evaluation of iCCA was performed using a Siemens Somatom Force spectral CT scanner (Siemens AG, Munich, Germany). The technical parameters for CT imaging were set as follows: tube voltage of 120 kVp, rotation time of 0.5 s per rotation, and reconstruction thickness of 5 mm. Different attenuation thresholds were used to delineate specific tissue components based on Hounsfield units (HUs). Visceral fat area (VFA) was identified using attenuation values ranging from −150 to −50 HU, while subcutaneous adipose tissue was segmented in the range of −190 to −30 HU. Skeletal muscle was identified and quantified using attenuation values ranging from −29 to 150 HUs, focusing on the muscle area at the level of the third lumbar vertebra (L3) (Figure 1).

The skeletal muscle index (SMI) was determined by normalizing the measured muscle area to the square of each patient’s height (cm^2^/m^2^). In addition, skeletal muscle radiation attenuation (SM-RA) in Hounsfield units (HUs) was recorded as an indicator of muscle density and myosteatosis. To accurately assess the cross-sectional areas of both skeletal muscle and adipose tissue at the L3 level, we used 3D Slicer software (version: 4.10.2; https://www.slicer.org/ (accessed on 3 March 2023)). All measurements were performed by the same investigator, who remained blinded to the clinical outcome of these patients. Obesity was defined as a BMI ≥ 25 kg/m^2^. Sarcopenia was characterized by a BMI < 25 kg/m^2^, with an SMI < 41 cm^2^/m^2^ for women and <43 cm^2^/m^2^ for men. In the context of a BMI ≥ 25 kg/m^2^, SMI < 53 cm^2^/m^2^. Myosteatosis was identified by a BMI < 25 kg/m^2^ and SM-RA < 41 HU. In individuals with a BMI ≥ 25 kg/m^2^, myosteatosis was indicated by SM-RA < 33 HU. Visceral obesity was indicated by a VFA ≥ 100 cm^2^. Sarcopenic obesity was defined as a BMI ≥ 25 kg/m^2^ with an SMI ≤ 38.5 cm^2^/m^2^ for women and 52.4 cm^2^/m^2^ for men. In that study, the intra-rater and inter-rater coefficient of variation was found to be 1.10% and 3.20% for SMI, 0.70% and 2.60% for VFA, and 1.20% and 2.40% for SM-RA, respectively.

### 2.3. Statistical Analysis

Statistical analyses were performed using IBM SPSS Statistics software, version 26.0. Both univariate and multivariate logistic regression analyses were performed to examine the interaction between different clinical parameters with a focus on complications and BC. The Kaplan–Meier method was used to assess overall survival (OS) and recurrence-free survival (RFS). Log-rank tests were used to determine the prognostic significance of BC and its associated clinical parameters. Cox regressions were performed to identify the prognostic factors associated with RFS and OS. Statistical significance was defined as *p*-values < 0.05, indicating the presence of significant differences.

## 3. Results

### 3.1. Patient Characteristics

This study included a cohort of 162 patients diagnosed with iCCA. All enrolled patients underwent preoperative CT imaging within three months prior to their respective surgical resections. The median age of the cohort was 66 years. Among those diagnosed with iCCA, a subset of 10 individuals (6.2%) experienced cholangitis. Most patients underwent major hepatic resections (89.5%). Subsequent pathological analysis unveiled an R1 rate of 9.9% accompanied by nodal metastases in 35.2% of the study cohort. The median follow-up for iCCA patients was 61 months, with a median RFS of 12 months. In addition, the median OS for patients in the iCCA group was also 22 months. Notably, a significant proportion of 68 patients (42.0%) showed postoperative complications rated Clavien–Dindo ≥ 3. Throughout the study period, the overall observed mortality rate was 68.7%. Comprehensive insight into the demographic data is provided in Table 1.

### 3.2. Associations between Body Composition and Clinical and Laboratory Features

In the iCCA group, 86 patients (53.1%) presented with obesity, 103 patients (63.6%) were classified as having sarcopenia, 86 patients (53.1%) exhibited myosteatosis, 101 patients (62.3%) displayed visceral obesity, and 25 patients (15.4%) demonstrated sarcopenic obesity (Table 1). To establish the relationship between these BC characteristics and clinical features, we conducted both univariate and multivariate logistic regression analyses. As complications were the focus of our analysis, we assessed overall complications according to the Clavien–Dindo Scale and infectious complications according to the Clavien–Dindo Scale, as well as liver failure, postoperative hemorrhage, and bile leakage according to the International Study Group of Liver Surgery (ISGLS) definitions [10,11,12,13].

Individuals with obesity exhibited associations with various perioperative parameters, e.g., diminished levels of alkaline phosphatase (AP) (*p* = 0.019) and platelet count (*p* = 0.048), lower pT category (*p* = 0.018), lower prothrombin time (*p* = 0.025), and shorter hospitalization (*p* = 0.003) as well as higher hemoglobin (*p* = 0.001) and albumin levels (*p* = 0.032). Further, a decreased likelihood of experiencing postoperative liver failure (*p* = 0.021), bile leakage (*p* = 0.004), severe postoperative complications (*p* = 0.006), and infectious complications (*p* = 0.018) showed significance in the univariate analysis. Statistically significant parameters were subsequently transferred to the multivariable analysis. Here, the presence of obesity was associated with higher hemoglobin levels (OR = 6.443, *p* < 0.001), shorter prothrombin time (OR = 0.229, *p* = 0.013), lower pT category (OR = 0.296, *p* = 0.026), and the male sex (OR = 0.431, *p* = 0.063).

ICCA patients with sarcopenia were predominantly female (*p* < 0.001) and showed lower levels of hemoglobin and a tendency for lympho-vascular invasion (LVI) (*p* = 0.080) according to univariate analysis. These patients were again subsequently analyzed using a multivariable analysis. Here, the female sex (OR = 2.263, *p* < 0.001) and the presence of LVI (OR = 2.307, *p* = 0.080) were statistically significant parameters.

In the subgroup of patients with myosteatosis various examined parameters showed statistical significance in the univariable logistic regression. Here, patients with myosteatosis were frequently women (*p* = 0.021) and elderly (*p* < 0.001) individuals. Furthermore, patients with myosteatosis exhibited reduced hemoglobin (*p* = 0.001) and albumin (*p* = 0.01) levels, an increased intraoperative transfusion rate of packed red blood cells (PRBCs) (*p* = 0.004), and a higher rate of lymphovascular invasion (LVI) (*p* = 0.043). Interestingly, patients in this group tended to receive no adjuvant therapy (*p* = 0.015). In the multivariate analysis, patients with myosteatosis were more likely to be female (OR = 2.636, *p* = 0.006), elderly (OR = 4.989, *p* = 0.001), display LVI (OR = 2.942, *p* = 0.041), and receive less adjuvant therapy (OR = 0.485, *p* = 0.099).

Individuals with visceral obesity were more commonly male (*p* < 0.001) and elderly (*p* = 0.004) according to the univariate logistic regression. Furthermore, this subgroup was associated with higher levels of hemoglobin (*p* = 0.004) and shorter hospitalization (*p* = 0.025). The multivariate analysis showed more male (OR = 0.247, *p* < 0.001) and elderly (OR = 3.577, *p* = 0.002) patients as well as reduced hemoglobin levels (OR = 3.265, *p* = 0.004) in this population.

Patients with sarcopenic obesity were also more frequently male (*p* < 0.001) and elderly (*p* = 0.008). These patients were also found to have reduced gamma-glutamyltransferase (GGT) (*p* = 0.040), elevated INR values (international normalized ratio) (*p* = 0.049), and less severe postoperative complications (*p* = 0.015). In the multivariate analysis, patients with sarcopenic obesity were more likely to be male (OR = 0.180, *p* = 0.004), elderly (OR = 4.007, *p* = 0.011), and show fewer postoperative complications (OR = 0.246, *p* = 0.015). More details about the univariate and multivariable analyses indicating a notable association between clinical and laboratory features and BC are displayed in Table 2. The detailed results of the univariate and multivariable analyses are shown in Supplementary Table S1.

### 3.3. Kaplan–Meier Analysis

**Figure 2:**
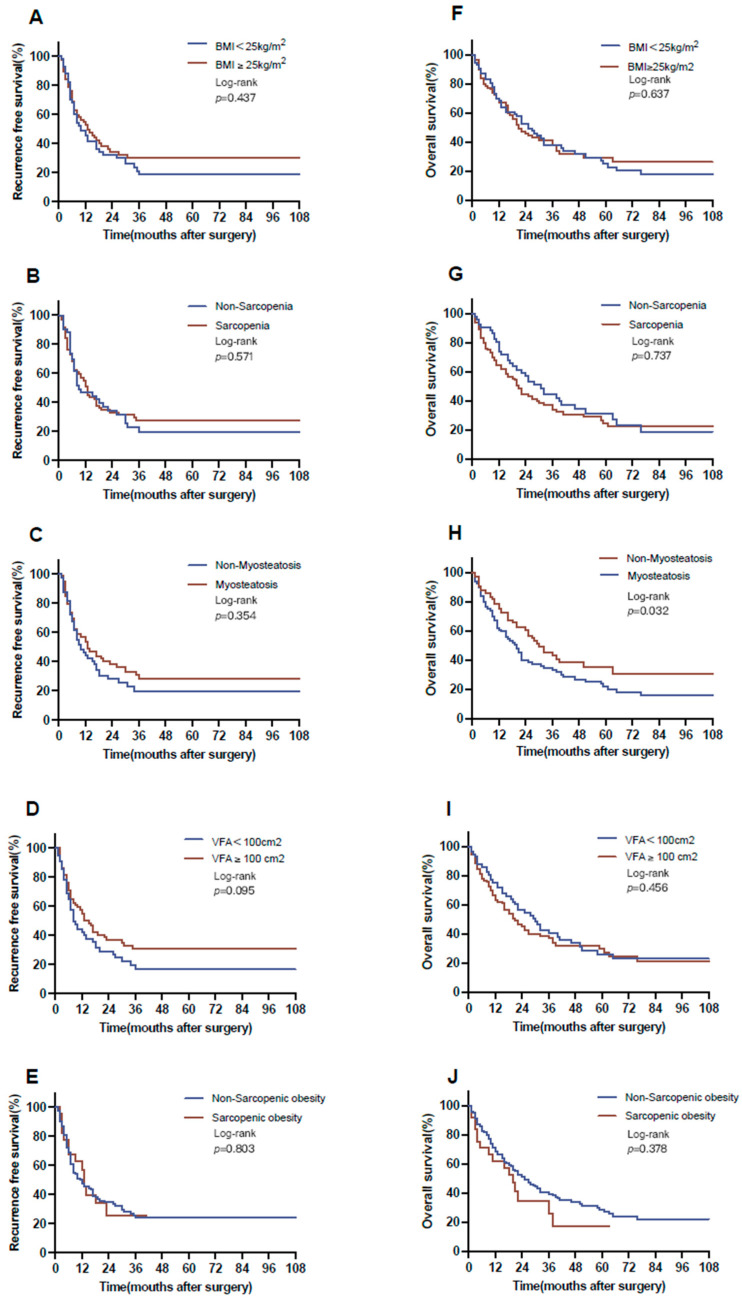
Overall and recurrence-free survival with respect to body composition. RFS for (**A**) BMI (BMI < 25 kg/m^2^ vs. BMI ≥ 25 kg/m^2^: 10 vs. 14 months), (**B**) sarcopenia (non-sarcopenia vs. sarcopenia: 8 vs. 13 months), (**C**) myosteatosis (non-myosteatosis vs. myosteatosis: 10 vs. 13 months), (**D**) visceral obesity (non-visceral obesity vs. visceral obesity: 8 vs. 13 months), and (**E**) sarcopenic obesity (non-sarcopenic obesity vs. sarcopenic obesity: 10 vs. 13 months). OS for (**F**) BMI (BMI < 25 kg/m^2^ vs. BMI ≥ 25 kg/m^2^: 22 vs. 21 months), (**G**) sarcopenia (non-sarcopenia vs. sarcopenia: 25 vs. 20 months), (**H**) myosteatosis (non-myosteatosis vs. myosteatosis: 29 vs. 17 months), (**I**) visceral obesity (non-visceral obesity vs. visceral obesity: 28 vs. 20 months) and (**J**) sarcopenic obesity (non-sarcopenic obesity vs. sarcopenic obesity: 22 vs. 20 months). BMI, body mass index; OS, over survival; RFS, recurrence-free survival.

Following a median follow-up of 61 months, the study cohort exhibited a median RFS of 12 months and a median OS of 22 months. No statistically significant differences were observed regarding various body composition parameters: non-obese versus obese (10 vs. 14 months, *p* = 0.437); non-sarcopenia versus sarcopenia (8 vs. 13 months, *p* = 0.571); non-myosteatosis versus myosteatosis (10 vs. 13 months, *p* = 0.354); non-visceral obesity versus visceral obesity (8 vs. 13 months, *p* = 0.120); and non-sarcopenic obesity versus sarcopenic obesity (10 vs. 13 months, *p* = 0.803) (Figure 2).

In terms of OS, patients with myosteatosis showed notably worse survival (17 months) in contrast to those in the non-myosteatosis group (29 months, *p* = 0.032 log-rank test). For all other BC parameters, no notable associations were observed (non-obese versus obese, 22 vs. 21 months, *p* = 0.627; non-sarcopenia versus sarcopenia, 25 vs. 20 months, *p* = 0.737; non-visceral obesity versus visceral obesity, 28 vs. 20 months, *p* = 0.345; and non-sarcopenic obesity versus sarcopenic obesity, 22 vs. 20 months, *p* = 0.378; Figure 2).

**Figure 3:**
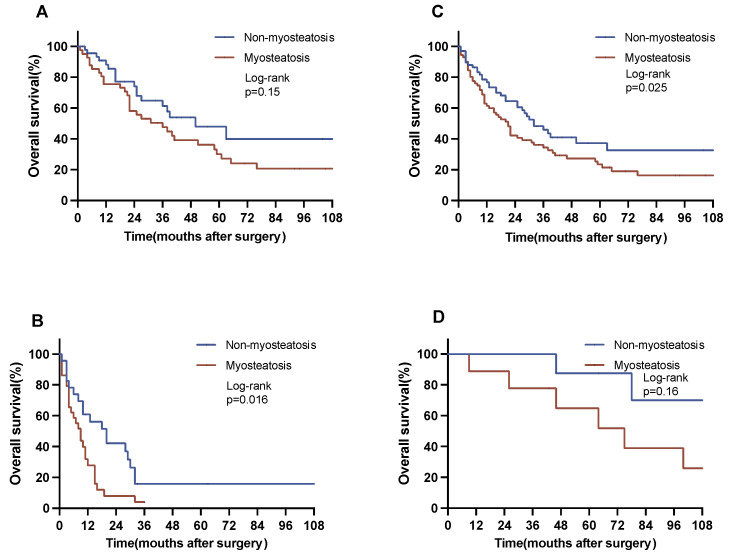
Overall survival in selected subgroups. The figure represents a subgroup analysis for OS. Subgroup analyses were conducted for the pN category and the R category. (**A**) Patients with no nodal metastases (N0, non-myosteatosis vs. myosteatosis: 50 vs. 31 months). (**B**) Patients with nodal metastases (N1, non-myosteatosis vs. myosteatosis: 18 vs. 7 months). (**C**) Patients with no residual tumor after resection (R0, non-myosteatosis vs. myosteatosis: 32 vs. 18 months). (**D**) Patients with residual tumor after resection (R1, non-myosteatosis vs. myosteatosis: 12 vs. 9 months).

A subgroup survival analysis was meticulously conducted, stratifying patients according to their residual tumor (R) classification, pT category, pN category, and body composition. Within the cohort of patients who underwent R0 resection, patients with myosteatosis exhibited a significantly reduced OS as opposed to their counterparts with no myosteatosis (18 vs. 32 months, *p* = 0.025 log-rank). Similarly, the subset of patients with lymph node metastases displayed differences with respect to myosteastosis: individuals with myosteatosis showed a substantial reduction in OS in comparison with patients without myosteatosis (7 vs. 18 months, *p* = 0.016) (Figure 3).

**Figure 4:**
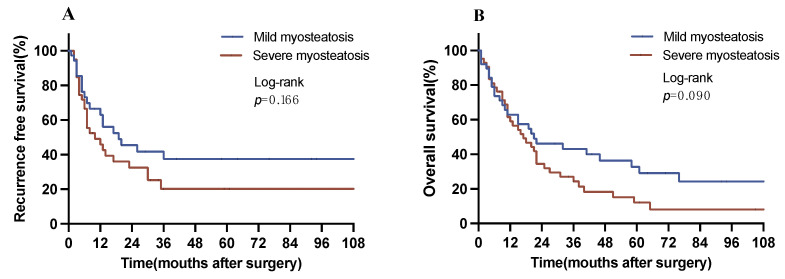
Overall and recurrence-free survival with respect to the degree of myosteatosis. (**A**) Recurrence-free survival in patients with myosteatosis (median RFS, mild vs. severe myosteatosis: 19 vs. 10 months). (**B**) Overall survival in patients with myosteatosis (median OS, mild vs. severe myosteatosis: 21 vs. 17 months). OS, overall survival. RFS, recurrence-free survival.

Another subgroup analysis was conducted patients with myosteatosis stratifying these individuals into patients with mild and severe myosteatosis based on the median cutoff for SM-RA, which is defined as SM-RA < 34 HUs for patients with BMI ≥ 25 kg/m^2^ and SM-RA < 27 HUs for patients with BMI < 25 kg/m^2^ for severe myosteatosis. Here, patients with severe myosteatosis exhibited a reduced RFS versus patients with mild myosteatosis (19 vs. 10 months, *p* = 0.166 log-rank). Similarly, individuals with severe myosteatosis showed a reduction in OS in comparison to patients with mild myosteatosis (21 vs. 17 months, *p* = 0.090 log-rank) (Figure 4). Both survival analyses displayed borderline significance (*p* = 0.166, *p* = 0.090).

### 3.4. Oncological Outcomes in iCCA

To investigate the relationship between BC and other clinical–pathological parameters with oncological outcomes, we conducted univariate and multivariate Cox regression analyses. In terms of RFS, neoadjuvant therapy (*p* = 0.006), AP (*p* = 0.007), C-reactive protein (CRP) (*p* < 0.001), GGT (*p* = 0.008), hemoglobin (*p* = 0.031), intraoperative PRBC (*p* = 0.004), intraoperative fresh frozen plasma (FFP) (*p* = 0.016), LVI (*p* < 0.001), R1 resection (*p* = 0.036), pN category (*p* < 0.001), hospitalization (*p* = 0.038), perioperative complications (*p* = 0.008), and infectious complications (*p* = 0.001) were found to be associated in the univariate analysis. All variables with *p*-values < 0.1 were included in the multivariate Cox regression model. Here, neoadjuvant therapy (HR = 3.607, *p* < 0.001), CRP (HR = 2.190, *p* = 0.008), and LVI (HR = 2.399, *p* = 0.004) were identified as independent predictors of RFS (Table 3).

Regarding OS, in the univariate analysis, age (*p* = 0.023), the American Society of Anesthesiologists (ASA) score (*p* = 0.023), neoadjuvant therapy (*p* = 0.019), AP (*p* = 0.002), CRP (*p* < 0.001), GGT (*p* = 0.008), hemoglobin (*p* = 0.007), intraoperative PRBC (*p* < 0.001), intraoperative FFP (*p* < 0.001), operative time (*p* = 0.001), LVI (*p* < 0.001), microvascular invasion(MVI) (*p* = 0.001), pT category (*p* = 0.016), pN category (*p* < 0.001), tumor grading (*p* < 0.001), intensive care unit (ICU) time (*p* = 0.044), hospitalization (*p* = 0.018), perioperative complications (*p* < 0.001), liver failure (*p* = 0.007), bile leak (*p* = 0.013), hemorrhage (*p* = 0.034), infection complications (*p* < 0.001), adjuvant therapy (*p* = 0.010), and myosteatosis (*p* = 0.036) were found to be significant. In the multivariate model, hemoglobin (HR = 0.410, *p* = 0.001), intraoperative FFP (HR = 2.546, *p* = 0.001), LVI (HR = 3.920, *p* < 0.001), MVI (HR = 1.034, *p* = 0.015), tumor grading (HR = 2.138, *p* = 0.004), perioperative complications (HR = 3.776, *p* = 0.002), and adjuvant therapy (HR = 0.273, *p* < 0.001) were identified as independent predictors of OS (Table 3).

## 4. Discussion

In this study, we observed prevalent changes in BC in patients with iCCA, where 53.1% had obesity, 63.2% had sarcopenia, 52.8% had myosteatosis, 62.3% had visceral obesity, and 15.3% had sarcopenic obesity. Notably, myosteatosis correlated with LVI and OS in iCCA patients. Interestingly, myosteatosis influenced OS in patients with lymph node metastasis, whereas its presence or absence had no statistically significant effect on OS in non-metastatic patients. However, no substantial associations were found between other body composition changes and markers of aggressive tumor biology, especially perioperative complications, which was one of the main aims of this study. To our knowledge, this study is the first to explore the postoperative prognostic significance of myosteatosis specifically in iCCA patients.

Myosteatosis is characterized by the infiltration of adipose tissue into muscle tissue. Numerous studies have demonstrated an association between myosteatosis and postoperative complications, the length of hospital stay, and mortality in several gastrointestinal cancers [14,15]. In our study, we found that myosteatosis was associated with LVI in patients and was also associated with overall survival (OS) in patients diagnosed with iCCA. LVI, as defined histologically, refers to the presence of tumor emboli within lymphatic or vascular channels or the invasion of lymphatic or vascular walls by cancer cells [16]. This phenomenon is particularly prominent in malignancies such as gastric, colorectal, and esophageal cancers, which are characterized by an increased incidence of LVI [17,18,19]. In addition, LVI is often an indicator of poor prognosis in general [17,18,19]. Furthermore, LVI is closely linked to lymph node metastasis and significantly impacts the overall survival of individuals with dCCA and pCCA [10,11]. Consistent with this trend, our study identified LVI as an independent prognostic factor for RFS and OS in individuals with iCCA. Based on our findings, we hypothesize that the influence of myosteatosis on OS in iCCA might be mediated by LVI.

Lymph node metastasis (LNM) is a common characteristic of advanced disease stages in iCCA and a strong predictor of poor prognosis in patients undergoing resection [12]. Studies have shown an association between BC and LNM in pancreatic ductal adenocarcinoma [13]. LNM occurs in approximately 39% of patients with iCCA who undergo lymph node dissection [20]. Furthermore, its occurrence is emerging as a strong independent prognostic risk factor in iCCA [21]. The underlying rationale for this phenomenon stems from the propensity of LNM to extend to distant lymph nodes, often beyond the confines of regional nodes [22]. Therefore, performing lymph node dissection alone is unlikely to result in a significant improvement in prognosis [23]. In our study cohort, 57 patients (35.2%) diagnosed with iCCA displayed LNM. Notably, among these individuals, those with myosteatosis experienced a significant decrease in OS compared with their non-myosteatosis counterparts. Given the presence of metastases in iCCA patients characterized by myosteatosis, we hypothesize that improvement in myosteatosis could potentially translate into improved prognosis for this specific subgroup. This is further supported by our observation that the degree of myosteatosis appears to be associated with the adverse effect on oncological outcomes in the subgroup of patients with myosteatosis in our analysis (Figure 4).

Evidence suggests that patients undergoing margin-negative surgical resection for iCCA have significantly improved overall survival [24]. A clear dichotomy emerges when comparing those with negative margins (R0 resection) to their counterparts with positive margins (R1 resection), with the latter being burdened by increased recurrence rates and compromised survival outcomes [25,26]. Intriguingly, our investigation revealed a remarkable finding: among individuals who underwent R0 resection, those with no myosteatosis had significantly longer OS, in contrast to those with concurrent myosteatosis. Notably, this disparity did not reach statistical significance within the R1 resection subgroup, possibly due to the strong effect of residual tumors in iCCA.

Among primary liver cancer, hepatocellular carcinoma (HCC) and iCCA predominate. Interestingly, sarcopenia has emerged as an autonomous predictor with marked potency, effectively prognosticating postoperative complications subsequent to surgical interventions targeting primary hepatic malignancies [27]. Sarcopenia is associated with major complications and prognosis after hepatectomy for HCC [28,29,30]. Skeletal muscle produces myofactors and pro-inflammatory cytokines, and myoblasts increase the levels of these pro-inflammatory cytokines [31,32]. Our hypothesis asserts that decreased muscle function and mass leads to an impaired immune response and an increased risk of HCC complications. The relationship between sarcopenia and postoperative major complications in pCCA remains a subject of debate. While certain studies negate any correlation between sarcopenia and severe complications in pCCA [33,34], others suggest a potential link, accentuating the ongoing discourse [35]. In contrast to HCC, our findings indicate that sarcopenia does not influence major postoperative complications in individuals with iCCA. Several potential explanations emerge as follows: 1. Sarcopenia correlates with systemic inflammatory and immune reactions; yet, HCC and iCCA might elicit distinct immune and inflammatory responses [36], which subsequently interact with the manifestations of sarcopenia. 2. HCC is commonly concomitant with cirrhosis, rendering patients more vulnerable to sarcopenia repercussions [37]. In contrast, iCCA patients seldom exhibit concomitant cirrhosis [38]. Currently, there are no effective therapeutic approaches to address myosteatosis in patients with iCCA. Recent research has suggested that enhancing perioperative and postoperative exercise regimens, including elements such as resistance training, could potentially improve postoperative recovery. The available evidence strongly suggests that omega-3 polyunsaturated fatty acids (PUFAs) play a constructive role in enhancing the capacity of mitochondrial oxidative phosphorylation in the context of human skeletal muscle [39]. This underscores their potential importance as a key intervention in the treatment of myosteatosis. In particular, the efficacy of eicosapentaenoic acid (EPA) and docosahexaenoic acid (DHA) in alleviating tumor-associated myosteatosis has been demonstrated in a preclinical model focused on colon cancer [40]. Expanding our molecular understanding of genes that may influence myosteatosis, sarcopenia and obesity may also help to improve the success of chemotherapies in iCCAs in the future. The animal model of Almasud et al. [39] interestingly showed that adipocyte-specific genes are activated in the musculature of tumor-bearing mice. In addition, treatment with genotoxic chemotherapeutic agents leads to a larger increase in senescent cells, which in turn mediate a proinflammatory milieu and promote muscle atrophy [41]. Personalized cancer therapy in the future would therefore ideally treat cancer and adverse body composition simultaneously. Interestingly, some genes that have been described to suppress the high body fat phenotype, hepatic steatosis, and potentially also myosteatosis are strongly downregulated in tumors because they are putative tumor-suppressor genes, such as *SFRP1*, *SFRP5*, and *DKK3* [42]. These genes often have functions in energy metabolism as well. For example, mice deficient in Sfrp1 exhibit increased adiposity and hepatic steatosis [43], and SFRP5 signaling has been shown to suppress non-alcoholic steatohepatitis [44]. Similarly, DKK3 was described to function as a negative regulator of insulin resistance and hepatic steatosis. Since tumor suppressor proteins lost in tumors cannot themselves be targeted, their function may be substituted with mimetic drugs [45]. This new concept involves the identification and validation of drug candidates that phenotypically mimic the lost tumor suppressor protein. Although still in the early stages of development, this concept may open up new modalities for targeted cancer therapy in iCCA as well.

Our study has several limitations. First, its retrospective nature, limited to a single institution, inevitably introduces selection bias. To mitigate this, multi-center and multi-country validation will be essential in the future. Second, the lack of an appropriate treatment strategy for patients with myosteatosis represents a significant clinical challenge. In addition, it should be recognized that certain pertinent data were not collected during the data collection process. These include aspects such as physical activity levels, nutritional status, and the use of certain medications with known effects on muscle physiology. To address these complexities, prospective, multi-institutional, randomized controlled trials should be designed and conducted in the future.

## 5. Conclusions

Our investigation revealed that BC had a minimal impact on both clinical and oncologic outcomes in patients with iCCA. However, a notable discovery emerged from our study, demonstrating a novel correlation between myosteatosis and LVI in iCCA patients, in addition to identifying myosteatosis as a prognostic indicator of OS. In addition, we identified LVI as an independent predictor of RFS and OS in iCCA patients. In the subset analysis of patients who underwent curative resection (R0) and those with regional lymph node involvement (N1), myosteatosis was associated with decreased OS. Despite these significant findings, no substantial associations were observed between BC and perioperative complications in iCCA. It is imperative to acknowledge that further experimental efforts are imperative to fully analyze and confirm these observations.

## Figures and Tables

**Table 1:** Patients’ characteristics.

Variables	iCCA (*n* = 162)
Demographics	
Gender, M/F (%)	76 (46.9)/86 (53.1)
Age (years)	66 (58–74)
ASA, *n* (%)	
I	4 (2.5)
II	64 (39.5)
III	87 (53.7)
IV	7 (4.3)
V	0 (0)
Cholangitis, *n* (%)	10 (6.2)
Portal vein embolization, *n* (%)	14 (8.6)
Preoperative chemotherapy, *n* (%)	21 (13.0)
Clinical chemistry	
AST (U/L)	33.0 (25.0–46.0)
ALT (U/L)	27.0 (18.0–48.0)
Albumin (g/L)	4.3 (4.0–4.6)
AP, U/L	118.0 (85.5–217.0)
CA199 (U/mL)	55.20 (20.00–274.40)
CRP (mg/L)	55.20 (20.00–274.40)
GGT (U/L)	109.0 (55.5–299.0)
Hemoglobin (g/dL)	13.0 (12.00–14.20)
INR	1.00 (0.96–1.07)
Platelet count (/nL)	247 (193–306)
Prothrombin time (%)	100 (89–107)
Total bilirubin (mg/dL)	0.46 (0.335–0.690)
Operative Data	
Intraoperative PRBC, *n* (%)	48 (29.6)
Intraoperative FFP, *n* (%)	56 (34.6)
Operative time (minutes)	300 (230–362)
Operative procedure, *n* (%)	
Bisegmentectomy	23 (14.1)
Hemihepatectomy	57 (35.0)
Extended hemihepatectomy	31 (19.0)
Trisectionectomy	20 (12.3)
Hepatoduodenoectomy	0 (0)
ALPPS	14 (8.6)
Others	18 (11.0)
Time to surgery, days	47 (30–89)
Pathological examination	
LVI, *n* (%)	33 (20.4)
MVI, *n* (%)	52 (32.1)
R1 resection, *n* (%)	16 (9.9)
pT category *n* (%)	
1	58 (35.8)
2	62 (38.3)
3	24 (14.8)
4	17 (10.5)
pN category, *n* (%)	
N0	91 (56.2)
N1	57 (35.2)
Tumor grading, *n* (%)	
G1	2 (1.2)
G2	94 (58.0)
G3	42 (25.9)
G4	5 (3.1)
Postoperative Data	
Intensive care, days	1 (1–1)
Hospitalization, days	13 (8–26)
Postoperative complications, *n* (%)	
No complications	56 (34.6)
Clavien–Dindo I	8 (4.9)
Clavien–Dindo II	30 (18.5)
Clavien–Dindo IIIa	34 (20.9)
Clavien–Dindo IIIb	13 (8.0)
Clavien–Dindo IVa	8 (5.0)
Clavien–Dindo IVb	1 (0.6)
Clavien–Dindo V	12 (7.4)
Liver failure, *n* (%)	
No failure	135 (82.8)
Grade A	13 (8.0)
Grade B	8 (4.9)
Grade C	7 (4.3)
Infection Clavien–Dindo	
No complications	89 (54.6)
Clavien–Dindo I	0 (0)
Clavien–Dindo II	30 (18.4)
Clavien–Dindo IIIa	30 (18.4)
Clavien–Dindo IIIb	4 (2.5)
Clavien–Dindo IVa	0 (0)
Clavien–Dindo IVb	1 (0.6)
Clavien–Dindo V	8 (4.9)
Bile leak, *n* (%)	
No	134 (82.2)
Grade A	3 (1.8)
Grade B	20 (12.3)
Grade C	6 (3.7)
Hemorrhage, *n* (%)	
No	150 (92.0)
Grade A	2 (1.2)
Grade B	2 (1.2)
Grade C	9 (5.5)
Oncologic Data	
Adjuvant chemotherapy, *n* (%)	56 (34.6)
Recurrence, *n* (%)	95 (58.6)
Median RFS, months (95% CI)	12 (8–16)
Median OS, months (95% CI)	22 (17–28)
Body composition	
BMI (kg/m^2^)	25.35 (22.61–29.27)
Visceral_fat area (cm^2^)	131.82 (64.67–216.76)
SMI (cm^2^/m^2^)	43.27 (38.93–50.24)
SM-RA (HU)	37.06 (28.97–41.72)
Obesity, *n* (%)	86 (53.1)
Sarcopenia, *n* (%)	103 (63.2)
Myosteatosis, *n* (%)	86 (52.8)
Visceral obesity, *n* (%)	101 (62.3)
Sarcopenic_obesity, *n* (%)	25 (15.3)

Note: Data are presented as median and interquartile range if not noted otherwise. ALPPS, associating liver partition with portal vein ligation for staged hepatectomy; ASA, American Society of Anesthesiology; AP, alkaline phosphatase; BMI, body mass index; CI, confidence interval; F, female; FFP, fresh frozen plasma; LVI, lymph vascular invasion; M, male; MVI, microvascular invasion; OS, overall survival; RFS, recurrence-free survival; SMI, skeletal muscle index; SM-RA, skeletal muscle radiation attenuation.

**Table 2:** Univariate and multivariable analyses of body composition and associated variables.

Outcome	Descriptives		Univariate Analysis		Multivariate Analysis	
BMI (kg/m^2^)	<25 (*n* = 77)	≥25 (*n* = 85)	OR (95% CI)	*p*=	OR (95% CI)	*p*=
Sex (male/female (%); ref = male)	30 (39.0)/47 (61.0)	46 (54.1)/39 (45.9)	0.541 (0.289–1.012)	0.055	0.431 (0.177–1.046)	0.063
ASA ((I/II)/(III/IV) (%); ref = I/II)	38 (49.4)/39 (50.6)	30 (35.3)/55 (64.7)	1.786 (0.951–3.356)	0.071	2.029 (0.785–5.243)	0.144
Cholangitis (no/yes (%); ref = no)	69 (89.6)/8 (10.4)	83 (97.6)/2 (2.4)	0.208 (0.043–1.011)	0.052	0.244 (0.017–3.406)	0.294
Albumin, g/L (≤4.2/>4.2 (%); ref = ≤4.2)	34 (44.2)/25 (32.5)	27 (31.8)/43 (50.6)	2.166 (1.069–4.387)	0.032	2.075 (0.937–4.595)	0.072
AP, U/L (≤100/>100 (%); ref = ≤100)	20 (26.0)/53 (68.8)	38 (44.7)/45 (52.9)	0.447 (0.228–0.875)	0.019	0.598 (0.249–1.437)	0.251
Hemoglobin, g/L (≤13/>13 (%); ref = ≤13)	51 (66.2)/25 (32.5)	25 (29.4)/59 (69.4)	4.814 (2.466–9.40)	0.001	**6.443 (2.538–16.359)**	**<0.001**
Platelet count (≤300/>300 (%); ref = ≤300)	50 (64.9)/26 (33.8)	67 (78.8)/17 (20.0)	0.488 (0.239–0.995)	0.048	0.517 (0.172–1.552)	0.240
Prothrombin time (≤110/>110 (%); ref = ≤110)	56 (72.7)/20 (26.0)	73 (85.9)/10 (11.8)	0.384 (0.166–0.884)	0.025	**0.229 (0.072–0.733)**	**0.013**
Intraoperative PRBC (no/yes (%); ref = no)	49 (63.6)/28 (36.4)	65 (76.5)/20 (23.5)	0.538 (0.272–1.066)	0.076	1.479 (0.396–5.525)	0.561
R1 resection ((R0/R2)/R1) (%); ref = R0/R2)	66 (85.7)/11 (14.3)	79 (92.9)/5 (5.9)	0.380 (0.126–1.148)	0.086	0.477 (0.095–2.400)	0.369
pT category (T1–2/T3–4 (%); ref = T1–T2)	50 (64.9)/26 (33.8)	70 (82.4)/15 (17.6)	0.412 (0.198–0.857)	0.018	**0.296 (0.101–0.864)**	**0.026**
Hospitalization, days (≤14/>14 (%); ref = ≤14)	32 (41.6)/45 (58.4)	55 (64.7)/30 (35.3)	0.388 (0.206–0.732)	0.003	0.925 (0.274–3.122)	0.900
Postoperative complications Clavien–Dindo ((0/I/II)/(III/IV/V) (%); ref = 0/I/II)	57 (74.0)/20 (26.0)	71 (83.5)/14 (16.5)	0.409 (0.216–0.775)	0.006	0.663 (0.145–3.037)	0.596
Liver failure (no/yes (%); ref = no)	58 (75.3)/19 (24.7)	76 (89.4)/9 (10.6)	0.361 (0.152–0.857)	0.021	0.548 (0.158–1.893)	0.341
Bile leak (no/yes (%); ref = no)	56 (72.7)/21 (27.3)	77 (90.6)/8 (9.4)	0.277 (0.114–0.671)	0.004	0.426 (0.123–1.467)	0.176
Hemorrhage (no/yes (%); ref = no)	70 (90.9)/7 (9.1)	79 (92.9)/6 (7.1)	0.759 (0.244–2.367)	0.635		
Infection Clavien–Dindo ((0/I/II)/(III/IV/V) (%); ref = 0/I/II)	66 (85.7)/10 (13.0)	82 (96.5)/3 (3.5)	0.421 (0.205–0.863)	0.018	0.896 (0.754–14.397)	0.113
Sarcopenia	No (*n* = 59)	Yes (*n* = 103)	OR (95% CI)	*p*=	OR (95% CI)	*p*=
Sex (male/female (%); ref = male)	40 (67.8)/19 (32.2)	36 (35.0)/67 (65.0)	3.918 (1.985–7.733)	<0.001	**2.263 (0.898–5.701)**	**<0.001**
Hemoglobin, g/L (≤13/>13 (%); ref = ≤13)	22 (37.3)/36 (61.0)	54 (52.4)/48 (46.6)	0.543 (0.281–1.049)	0.069	0.560 (0.270–1.160)	0.119
LVI (no/yes (%); ref = no)	49 (83.1)/8 (13.6)	70 (68.0)/25 (24.3)	2.187 (0.911–5.252)	0.080	2.307 (0.906–5.874)	0.080
Postoperative complications Clavien–Dindo ((0/I/II )/(III/IV/V) (%); ref = 0/I/II)	47 (79.7)/12 (20.3)	81 (78.6)/22 (21.4)	0.874 (0.458–1.669)	0.683		
Liver failure (no/yes (%); ref = no)	47 (79.7)/12 (20.3)	87 (84.5)/16 (15.5)	0.720 (0.315–1.649)	0.438		
Bile leak (no/yes (%); ref = no)	46 (78.0)/13 (22.0)	87 (84.5)/16 (15.5)	0.651 (0.288–1.469)	0.301		
Hemorrhage (no/yes (%); ref = no)	55 (93.2)/4 (6.8)	94 (91.3)/9 (8.7)	1.316 (0.387–4.477)	0.660		
Infection Clavien–Dindo ((0/I/II)/(III/IV/V) (%); ref = 0/I/II)	54 (91.5)/5 (8.5)	94 (91.3)/8 (7.8)	0.740 (0.362–1.511)	0.408		
Myosteatosis	No (*n* = 76)	Yes (*n* = 86)	OR (95% CI)	*p*=	OR (95% CI)	*p*=
Sex (male/female (%); ref = male)	43 (56.6)/33 (43.4)	33 (38.4)/53 (61.6)	2.093 (1.117–3.922)	0.021	**3.636 (1.447–9.132)**	**0.006**
Age (≤65/>65 years (%); ref = ≤65)	50 (65.8)/26 (34.2)	30 (34.9)/56 (65.1)	3.590 (1.876–6.870)	<0.001	**4.989 (1.904–13.068)**	**0.001**
Albumin, g/L (≤4.2/>4.2 (%); ref = ≤4.2)	22 (28.9)/40 (52.6)	39 (45.3)/28 (32.6)	0.395 (0.194–804)	0.010	0.817 (0.318–2.096)	0.674
Hemoglobin, g/L (≤13/>13 (%); ref = ≤13)	25 (32.9)/50 (65.8)	51 (59.3)/34 (39.5)	0.333 (0.175–0.637)	0.001	0.535 (0.230–1.242)	0.145
Total bilirubin, mg/dL (≤1/>1 (%); ref = ≤1)	69 (90.8)/6 (7.9)	69 (80.2)/16 (18.6)	2.667 (0.985–7.219)	0.054	2.141 (0.637–7.196)	0.218
Intraoperative PRBC (no/yes (%); ref = no)	62 (81.6)/14 (18.4)	52 (60.5)/34 (39.5)	2.896 (1.405–5.969)	0.004	1.594 (0.594–4.277)	0.354
LVI (no/yes (%); ref = no)	60 (78.9)/10 (13.2)	59 (68.6)/23 (26.7)	2.339 (1.025–5.336)	0.043	**2.942 (1.047–8.268)**	**0.041**
Postoperative complications Clavien–Dindo ((0/I/II)/(III/IV/V) (%); ref = 0/I/II)	47 (61.8)/29 (38.2)	47 (54.7)/39 (45.3)	1.345 (0.718–2.520)	0.355		
Liver failure (no/yes (%); ref = no)	66 (86.8)/10 (13.2)	68 (79.1)/18 (20.9)	1.747 (0.751–4.063)	0.195		
Bile leak (no/yes (%); ref = no)	62 (81.6)/14 (18.4)	71 (82.6)/15 (17.4)	0.936 (0.419–2.091)	0.871		
Hemorrhage (no/yes (%); ref = no)	72 (94.7)/4 (5.3)	77 (89.5)/9 (10.5)	2.104 (0.621–7.132)	0.232		
Infection Clavien–Dindo ((0/I/II)/(III/IV/V) (%); ref = 0/I/II)	72 (94.7)/3 (3.9)	76 (88.4)/10 (11.6)	1.004 (0.499–2.022)	0.991		
Adjuvant therapy (no/yes (%); ref = no)	39 (51.3)/33 (43.4)	62 (72.1)/23 (26.7)	0.438 (0.225–0.854)	0.015	0.485 (0.205–1.146)	0.099
VFA (cm^2^)	<100 (*n* = 61)	≥100 (*n* = 101)	OR (95% CI)	*p*=	OR (95% CI)	*p*=
Sex (male/female (%); ref = male)	13 (21.3)/48 (78.7)	63 (62.4)/38 (37.6)	0.163 (0.078–0.340)	<0.001	**0.247 (0.114–0.536)**	**<0.001**
Age (≤65/>65 years (%); ref = ≤65)	39 (63.9)/22 (36.1)	41 (40.6)/60 (59.4)	2.594 (1.346–5.001)	0.004	**3.577 (1.592–8.035)**	**0.002**
Hemoglobin, g/L (≤13/>13 (%); ref = ≤13)	37 (60.7)/22 (36.1)	39 (38.6)/62 (61.4)	2.674 (1.378–5.186)	0.004	**3.265 (1.455–7.325)**	**0.004**
INR (≤1/>1 (%); ref = ≤1)	36 (59.0)/23 (37.7)	47 (46.5)/53 (52.5)	1.765 (0.918–3.395)	0.089	1.310 (0.603–2.844)	0.495
Hospitalization, days (≤14/>14 (%); ref = ≤14)	26 (42.6)/35 (57.4)	61 (60.4)/40 (39.6)	0.487 (0.255–0.929)	0.029	0.570 (0.260–1.251)	0.161
Postoperative complications Clavien–Dindo ((0/I/II)/(III/IV/V) (%); ref = 0/I/II)	45 (73.8)/16 (26.2)	63 (62.4)/38 (37.6)	0.614 (0.323–1.167)	0.136		
Liver failure (no/yes (%); ref = no)	47 (77.0)/14 (23.0)	87 (86.1)/14 (13.9)	0.540 (0.238–1.228)	0.142		
Bile leak (no/yes (%); ref = no)	46 (75.4)/15 (24.6)	87 (86.1)/14 (13.9)	0.493 (0.219–1.111)	0.088	0.636 (0.229–1.765)	0.385
Hemorrhage (no/yes (%); ref = no)	55 (90.2)/6 (9.8)	94 (93.1)/7 (6.9)	0.683 (0.218–2.135)	0.512		
Infection Clavien–Dindo ((0/I/II)/(III/IV/V) (%); ref = 0/I/II)	42 (68.9)/18 (29.5)	76 (75.2)/25 (24.8)	0.758 (0.371–1.546)	0.445		
Sarcopenic_obesity	No (*n* = 137)	Yes (*n* = 25)	OR (95% CI)	*p*=	OR (95% CI)	*p*=
Sex (male/female (%); ref = male)	55 (40.1)/82 (59.9)	21 (84.0)/4 (16.0)	0.128 (0.042–0.393)	<0.001	**0.180 (0.056–0.582)**	**0.004**
Age (≤65/>65 years (%); ref = ≤65)	74 (54.0)/63 (46.0)	6 (24.0)/19 (76.0)	3.720 (1.400–9.885)	0.008	**4.007 (1.375–11.674)**	**0.011**
AP, U/L (≤100/>100 (%); ref = ≤100)	45 (32.8)/86 (62.8)	13 (52.0)/12 (48.0)	0.483 (0.204–1.145)	0.099	1.009 (0.292–3.479)	0.989
GGT,U/L (≤100/>100 (%); ref = ≤100)	59 (43.1)/72 (52.6)	17 (68.0)/8 (32.0)	0.386 (0.156–0.956)	0.040	0.405 (0.134–1.225)	0.110
INR (≤1/>1 (%); ref = ≤1)	75 (54.7)/60 (43.8)	8 (32.0)/16 (64.0)	2.500 (1.002–6.236)	0.049	1.247 (0.419–3.714)	0.691
Platelet count (≤300/>300 (%); ref = ≤300)	95 (69.3)/40 (29.2)	22 (88.0)/3 (12.0)	0.324 (0.092–1.144)	0.080	0.779 (0.184–3.299)	0.735
MVI (no/yes (%); ref = no)	82 (59.9)/48 (35.0)	21 (84.0)/4 (16.0)	0.325 (0.105–1.004)	0.051	0.438 (0.128–1.498)	0.188
Hospitalization, days (≤14/>14 (%); ref = ≤14)	69 (50.4)/68 (49.6)	18 (72.0)/7 (28.0)	0.395 (0.155–1.005)	0.051	2.382 (0.453–12.525)	0.306
Postoperative complications Clavien–Dindo ((0/I/II)/(III/IV/V) (%); ref = 0/I/II)	74 (54.0)/63 (46.0)	20 (80.0)/5 (20.0)	0.294 (0.104–0.827)	0.020	**0.246 (0.080–0.760)**	**0.015**
Liver failure (no/yes (%); ref = no)	111 (81.0)/26 (19.0)	23 (92.0)/2 (8.0)	0.371 (0.082–1.675)	0.197		
Bile leak (no/yes (%); ref = no)	109 (79.6)/28 (20.4)	24 (96.0)/1 (4.0)	0.162 (0.021–1.251)	0.081	0.363 (0.036–3.718)	0.394
Hemorrhage (no/yes (%); ref = no)	124 (90.5)/13 (9.5)	130 (81.3)/30 (18.8)	0 (0–0)	0.999		
Infection Clavien–Dindo ((0/I/II)/(III/IV/V) (%); ref = 0/I/II)	96 (70.1)/40 (29.2)	22 (88.0)/3 (12.0)	0.327 (0.093–1.155)	0.083	0.894 (0.096–8.339)	0.921

Multiple variables were associated with altered body composition. This reduced table only shows variables with a *p*-value < 0.1 and all measures of postoperative complications in the univariate analysis. Variables displaying a *p*-value < 0.1 in the univariate analysis were transferred into a multivariable logistic regression model. Bold letters indicate statistical significance. The univariate analysis regarding all variables is displayed in Supplementary Table S1. Abbreviations: AP, alkaline phosphatase; ASA, American Society of Anesthesiologists; BMI, body mass index; GGT, gamma-glutamyl transferase; INR, international normalized ratio; LVI, lymph vascular invasion; MVI, microvascular invasion.

**Table 3:** Recurrence-free survival and overall survival in intrahepatic cholangiocarcinoma.

	RFS				OS			
	Univariable		Multivariable		Univariable		Multivariable	
Variables	HR (95%Cl)	*p*	HR (95%Cl)	*p*	HR (95%Cl)	*p*	HR (95%CI)	*p*
Sex (male = 1)	1.034 (0.688–1.554)	0.871			0.730 (0.502–1.602)	0.100		
Age, years (≤65 = 1)	0.769 (0.510–1.159)	0.209			1.544 (1.061–2.247)	0.023	1.564 (0.894–2.736)	0.117
ASA (I/II = 1)	1.131 (0.753–1.699)	0.554			0.730 (0.502–1.602)	0.100		
Cholangitis (no = 1)	0.266 (0.065–1.085)	0.065	0.238 (0.057–1.044)	0.049	1.544 (1.061–2.247)	0.023	1.077 (0.414–2.804)	0.879
PVE (no = 1)	1.496 (0.750–2.987)	0.253			1.714 (0.937–3.135)	0.080	1.878 (0.746–4.727)	0.181
Neoadjuvant therapy (no = 1)	2.271 (1.270–4.061)	0.006	3.607 (1.758–7.402)	<0.001	1.907 (1.112–3.270)	0.019	1.771 (0.554–5.659)	0.575
AST, U/L (≤40 = 1)	1.264 (0.829–1.927)	0.277			1.108 (0.751–1.633)	0.606		
ALT, U/L (≤40 = 1)	1.186 (0.743–1.892)	0.474			1.090 (0.705–1.685)	0.697		
AP, U/L (≤100 = 1)	1.829 (1.177–2.842)	0.007	1.218 (0.652–2.278)	0.536	1.958 (1.276–3.005)	0.002	1.193 (0.589–2.416)	0.624
CRP, mg/L (≤8.2 = 1)	2.319 (1.513–3.555)	<0.001	2.190 (1.232–3.894)	0.008	2.308 (1.379–3.012)	<0.001	1.747 (0.815–3.746)	0.152
GGT, U/L (≤100 = 1)	1.751 (1.155–2.654)	0.008	1.124 (0.620–2.039)	0.700	1.691 (1.147–2.493)	0.008	1.189 (0.612–2.308)	0.610
Hemoglobin, g/dL (≤13 = 1)	0.637 (0.423–0.960)	0.031	0.901 (0.515–1.576)	0.715	0.595 (0.409–0.868)	0.007	0.410 (0.243–0.693)	0.001
INR (≤1 = 1)	1.497 (0.990–2.265)	0.056	1.603 (0.970–2.648)	0.065	1.380 (0.946–2.013)	0.095	1.030 (0.573–1.851)	0.921
Platelet count, L/nL (≤300 = 1)	1.499 (0.965–2.330)	0.072	1.008 (0.587–1.731)	0.977	0.901 (0.591–1.374)	0.629		
Prothrombin time (≤110 = 1)	0.761 (0.448–1.292)	0.312			0.643 (0.386–1.070)	0.089	0.804 (0.348–1.855)	0.609
Bilirubin, mg/dL (≤1 = 1)	0.789 (0.420–1.483)	0.462			1.229 (0.740–2.041)	0.425		
Intraoperative PRBC (No = 1)	1.906 (1.235–2.942)	0.004	1.379 (0.756–2.515)	0.295	2.001 (1.366–2.930)	<0.001	0.726 (0.307–1.714)	0.465
Intraoperative FFP (No = 1)	1.687 (1.104–2.578)	0.016	1.274 (0.785–2.066)	0.327	1.986 (1.365–2.890)	<0.001	2.546 (1.449–4.473)	0.001
Operative time, min (≤360 = 1)	1.562 (0.993–2.459)	0.054	1.028 (0.574–1.839)	0.926	1.988 (1.324–2.986)	0.001	1.680 (0.883–3.199)	0.114
Time to surgery, days (≤30 = 1)	0.704 (0.451–1.097)	0.120			1.022 (0.698–1.497)	0.911		
LVI (no = 1)	2.600 (1.569–4.309)	<0.001	2.399 (1.316–4.373)	0.004	3.416 (2.208–5.285)	<0.001	3.920 (2.197–6.992)	< 0.001
MVI (no = 1)	1.027 (0.956–1.103)	0.470			1.038 (1.016–1.061)	0.001	1.034 (1.006–1.062)	0.015
R1 resection (R0/Rx = 1)	1.992 (1.045–3.764)	0.036	1.290 (0.524–3.174)	0.579	1.768 (0.984–3.176)	0.056	1.290 (0.455–3.659)	0.632
pT category (T1/T2 = 1)	1.341 (0.836–2.152)	0.224			1.674 (1.099–2.550)	0.016	1.291 (0.684–2.435)	0.431
pN category (N0 = 1)	2.871 (1.828–4.511)	<0.001	1.525 (0.809–2.878)	0.192	3.320 (2.198–5.015)	<0.001	1.195 (0.487–2.934)	0.697
Tumor grading (G1/G2 = 1)	1.441 (0.895–2.318)	0.133			2.118 (1.389–3.230)	<0.001	2.138 (1.272–3.594)	0.004
ICU time, days (≤1 = 1)	1.122 (0.669–1.879)	0.663			1.579 (1.013–2.459)	0.044	1.052 (0.502–2.203)	0.894
Hospitalization, days (≤14 = 1)	1.023 (2.308–)	0.038	1.036 (0.541–1.985)	0.915	1.567 (1.079–2.277)	0.018	1.024 (0.520–2.019)	0.944
Perioperative complications (Clavien–Dindo 0/I/II = 1)	1.7533 (1.159–2.653)	0.008	1.631 (0.900–2.957)	0.107	2.230 (1.532–3.247)	<0.001	3.776 (1.617–8.817)	0.002
Liver failure (no = 1)	1.597 (0.902–2.830)	0.108			1.815 (1.153–2.858)	0.010	1.144 (0.611–2.142)	0.674
Bile leak (no = 1)	1.377 (0.811–2.338)	0.236			1.809 (1.135–2.883)	0.013	1.082 (0.483–2.420)	0.849
Hemorrhage (No = 1)	0.928 (0.377–2.286)	0.871			2.304 (1.055–3.919)	0.034	1.850 (0.668–5.122)	0.236
Infection complications (Clavien–Dindo 0/I/II = 1)	2.256 (1.421–3.580)	0.001	1.658 (0.955–2.878)	0.073	2.438 (1.624–3.660)	<0.001	1.545 (0.655–3.641)	0.320
Adjuvant therapy (no = 1)	1.145 (0.759–1.728)	0.518			0.620 (0.406–0.948)	0.010	0.273 (0.149–0.500)	<0.001
BMI, kg/m^2^ (≤25 = 1)	0.855 (0.571–1.282)	0.450			0.913 (0.629–1.326)	0.632		
Visceral_fat area (≤100 = 1)	0.731 (0.487–1.098)	0.131			1.067 (0.726–1.568)	0.741		
Sarcopenia (no = 1)	0.890 (0.587–1.349)	0.586			1.509 (1.027–2.217)	0.036	1.293 (0.640–2.613)	0.474
Myosteatosis (no = 1)	0.829 (0.553–1.245)	0.367			1.200 (0.817–1.762)	0.352		
Sarcopenic_obesity (no = 1)	0.932 (0.528–1.646)	0.808			1.267 (0.742–2.165)	0.386		

Multiple variables were associated with recurrence-free and overall survival. Variables displaying a *p*-value < 0.1 in the univariate Cox regression were transferred into a multivariable Cox regression model. The table heading is shown in grey background. ALT, alanine aminotransferase; ASA, American Society of Anesthesiologists; AST, aspartate aminotransferase; BMI, body mass index; CRP, C-reactive protein; FFP, fresh frozen plasma; GGT, gamma-glutamyl transferase; HR, hazard ratio; ICU, intensive care unit; INR, international normalized ratio; LVI, lymph vascular invasion; MVI, microvascular invasion; PVE, portal vein embolization; RFS, recurrence-free survival.

## Data Availability

Data and materials supporting the results or analyses presented in this paper are available upon reasonable request.

## Supplementary Materials

The following supporting information can be downloaded at: https://www.mdpi.com/article/10.3390/jcm12247747/s1, Table S1: Univariate and multivariate analysis of associations between body composition and clinical features in cholangiocarcinoma.
