# Supplementary material for: Impact of Altered Body Composition on Clinical and Oncological Outcomes in Intrahepatic Cholangiocarcinoma
Source: J Clin Med. 2023 Dec 18;12(24):7747. doi: 10.3390/jcm12247747 (PMC10744221; doi:10.3390/jcm12247747)
Supplement: Supplementary file 1 [file jcm-12-07747-s001.zip › jcm-2730016-supplementary.pdf]

Supplementary Table S1 Univariate and multivariate analysis of associations between body composition and clinical features in cholangiocarcinoma.

Outcome	Desriptives		Univarite analysis		Multivariate Analysis	
BMI(kg/m^2)	< 25(n=77)	≥25(n=85)	OR (95% CI)	p=	OR (95% CI)	p=
Sex (male/female(%); ref=male)	30(39.0)/47(61.0)	46(54.1)/39(45.9)	0.541(0.289-1.012)	0.055	0.431(0.177-1.046)	0.063
Age (≤65/ >65 years(%); ref=≤65)	35(45.5)/42(54.5)	45(52.9)/40(47.1)	0.741(0.399-1.375)	0.342		
ASA ((I/II)/(III/IV) (%);ref= I/II)	38(49.4)/39(50.6)	30(35.3)/55(64.7)	1.786(0.951-3.356)	0.071	0.244(0.017-3.406)	0.144
Cholangitis (No/Yes(%);ref=No)	69(89.6)/8(10.4)	83(97.6)/2(2.4)	0.208(0.043-1.011)	0.052	0.244(0.017-3.406)	0.294
PVE (No/Yes(%);ref=No)	68(88.3)/9(11.7)	80(94.1)/5(5.9)	0.472(0.151-1.477)	0.197		
Neoadjuvant therapy ((No/Yes(%);ref=No)	66(85.7)/11(14.3)	75(88.2)/10(11.8)	0.800(0.319-2.003)	0.634		
AST, U/L (≤40/ >40(%); ref=≤40)	52(67.5)/25(32.5)	58(68.2)/27(31.8)	0.968(0.500-1.874)	0.924		
ALT, U/L (≤40/ >40(%); ref=≤40)	46(59.7)/18(23.4)	51(60.0)/25(29.4)	1.253(0.607-2.587)	0.543		
Albumin,g/L (≤4.2/ >4.2(%); ref=≤4.2)	34(44.2)/25(32.5)	27(31.8)/43(50.6)	2.166(1.069-4.387)	0.032	2.075(0.937-4.595)	0.567
AP,U/L (≤100/ >100(%); ref=≤100)	20(26.0)/53(68.8)	38(44.7)/45(52.9)	0.447(0.228-0.875)	0.019	0.598(0.249-1.437)	0.465
CRP,mg/l (≤8.2/ >8.2(%); ref=≤8.2)	39(50.6)/33(42.9)	50(58.8)/31(36.5)	1.254(0.704-2.235)	0.344		
GGT,U/L (≤100/ >100(%); ref=≤100)	34(44.2)/39(50.6)	42(49.4)/41(48.2)	0.851(0.453-1.598)	0.616		
Hemoglobin, g/L (≤13/ >13(%); ref=≤13)	51(66.2)/25(32.5)	25(29.4)/59(69.4)	4.814(2.466-9.40)	0.001	**6.443(2.538-16.359)**	**< 0.001**
INR (≤1/>1(%); ref=≤1)	43(55.8)/33(42.9)	40(47.1)/43(50.6)	1.401(0.749-2.618)	0.291		

Platelet count<br>(≤300/ >300(%); ref=≤300)	50(64.9)/26(33.8)	67(78.8)/17(20.0)	0.488(0.239-0.995)	0.048	0.517(0.172-1.552)	0.240
Prothrombin time<br>(≤110/ >110(%); ref=≤110)	56(72.7)/20(26.0)	73(85.9)/10(11.8)	0.384(0.166-0.884)	0.025	**0.229(0.072-0.733)**	**0.013**
Total bilirubin,mg/dl<br>(≤1/ >1(%); ref=≤1)	62(80.5)/14(18.2)	76(89.4)/8(9.4)	0.466(0.184-1.183)	0.108		
Intraop PRBC<br>(No/Yes(%);ref=No)	49(63.6)/28(36.4)	65(76.5)/20(23.5)	0.538(0.272-1.066)	0.076	1.479(0.396-5.525)	0.561
Intraop FFP<br>((No/Yes(%);ref=No)	50(64.9)/27(35.1)	56(65.9)/29(34.1)	0.959(0.502-1.834)	0.899		
Operative time,minutes<br>(≤360/ >360(%); ref=≤360)	57(74.0)/20(26.0)	58(68.2)/27(31.8)	1.327(0.669-2.630)	0.418		
Time to surgery,days<br>(≤30/ >30(%); ref=≤30)	17(22.1)/52(67.5)	20(23.5)/57(67.1)	0.932(0.441-1.968)	0.853		
LVI<br>(No/Yes(%);ref=No)	60(77.9)/13(16.9)	59(69.4)/20(23.5)	1.565(0.713-3.431)	0.264		
MVI<br>(No/Yes(%);ref=No)	45(58.4)/28(36.4)	58(68.2)/24(28.2)	0.655(0.340-1.300)	0.233		
R1 resection<br>((R0/R2)/R1) (%); ref=R0/R2)	66(85.7)/11(14.3)	79(92.9)/5(5.9)	0.380(0.126-1.148)	0.086	0.477(0.095-2.400)	0.369
pT category<br>(T1-2/T3-4(%);ref=T1-T2)	50(64.9)/26(33.8)	70(82.4)/15(17.6)	0.412(0.198-0.857)	0.018	**0.296(0.101-0.864)**	**0.026**
pN category<br>(N0/N1(%);ref=N0)	42(54.5)/30(39.0)	49(57.6)/27(31.8)	0.771(0.397-1.498)	0.443		
Tumor grading<br>((G1/G2)/(G3/G4) (%);ref= G1/G2)	43(55.8)/25(32.5)	52(61.2)/23(27.1)	0.761(0.379-1.525)	0.441		
ICU time,days<br>(≤1/>1(%),ref=1)	59(76.6)/18(23.4)	68(80.0)/17(20.0)	0.819(0.387-1.733)	0.602		
Hospitalization,days<br>(≤14/>14(%); ref=≤14)	32(41.6)/45(58.4)	55(64.7)/30(35.3)	0.388(0.206-0.732)	0.003	0.925(0.274-3.122)	0.900
Postoperative complications Clavien-Dindo((0/I/II)/(III/IV/V) (%);ref= 0/I/II)	57(74.0)/20(26.0)	71(83.5)/14(16.5)	0.409(0.216-0.775)	0.006	0.663(0.145-3.037)	0.596

Liver failure (No/Yes(%);ref=No)	58(75.3)/19(24.7)	76(89.4)/9(10.6)	0.361(0.152-0.857)	0.021	0.548(0.158-1.893)	0.341
Bile leak, (No/Yes(%);ref=No)	56(72.7)/21(27.3)	77(90.6)/8(9.4)	0.277(0.114-0.671)	0.004	0.426(0.123-1.467)	0.176
Hemorrhage, (No/Yes(%);ref=No)	70(90.9)/7(9.1)	79(92.9)/6(7.1)	0.759(0.244-2.367)	0.635		
Infection Clavien-Dindo((0/I/II)/(III/IV/V)(%);ref= 0/I/II)	66(85.7)/10(13.0)	82(96.5)/3(3.5)	0.421(0.205-0.863)	0.018	0.896(0.754-14.397)	0.113
Adjuvant therapy (No/Yes(%);ref=No)	52(67.5)/22(28.6)	49(57.6)/34(40.0)	1.640(0.845-3.183)	0.144		
Sarcopenia	No(n=59)	Yes(n=103)	OR (95% CI)	p=	OR (95% CI)	p=
Sex (male/female(%); ref=male)	40(67.8)/19(32.2)	36(35.0)/67(65.0)	3.918(1.985-7.733)	< 0.001	**2.263(0.898-5.701)**	**< 0.001**
Age (≤65/ >65 years(%); ref=≤65)	29(49.2)/30(50.8)	51(49.5)/52(50.5)	0.986(0.520-1.869)	0.965		
ASA ((I/II)/(III/IV) (%);ref= I/II)	24(40.7)/35(59.3)	44(42.7)/59(57.3)	0.919(0.480-1.761)	0.800		
Cholangitis (No/Yes(%);ref=No)	55(93.2)/4(6.8)	97(94.2)/6(5.8)	0.851(0.230-3.145)	0.808		
PVE (No/Yes(%);ref=No)	55(93.2)/4(6.8)	93(90.3)/10(9.7)	1.478(0.442-4.941)	0.525		
Neoadjuvant therapy ((No/Yes(%);ref=No)	54(91.5)/5(8.5)	87(84.5)/16(15.5)	1.986(0.688-5.733)	0.205		
AST, U/L (≤40/ >40(%); ref=≤40)	40(67.8)/19(32.2)	70(68.0)/33(32.0)	0.992(0.500-1.969)	0.983		
ALT, U/L (≤40/ >40(%); ref=≤40)	33(55.9)/19(32.2)	64(62.1)/24(23.3)	0.651(0.313-1.357)	0.252		
Albumin,g/L (≤4.2/ >4.2(%); ref=≤4.2)	21(35.6)/25(42.4)	40(38.8)/43(41.7)	0.903(0.438-1.860)	0.782		
AP,U/L (≤100/ >100(%); ref=≤100)	20(33.9)/37(62.7)	38(36.9)/61(57.2)	0.868(0.440-1.710)	0.682		
CRP,mg/l (≤8.2/ >8.2(%); ref=≤8.2)	32(54.2)/24(40.7)	57(55.3)/40(38.8)	0.892(0.461-1.724)	0.733		
GGT,U/L	23(39.0)/34(57.6)	53(51.5)/46(44.7)	0.587(0.303-1.136)	0.114		

(≤100/ >100(%); ref=≤100)						
Hemoglobin, g/L<br>(≤13/ >13(%); ref=≤13)	22(37.3)/36(61.0)	54(52.4)/48(46.6)	0.543(0.281-1.049)	0.069	0.560(0.270-1.160)	0.119
INR<br>(≤1/>1(%); ref=≤1)	29(49.2)/29(49.2)	54(52.4)/47(45.6)	0.870(0.456-1.661)	0.674		
Platelet count<br>(≤300/ >300(%); ref=≤300)	46(78.0)/12(20.3)	71(68.9)/31(30.1)	1.674(0.781-3.588)	0.186		
Prothrombin time<br>(≤110/ >110(%); ref=≤110)	46(78.0)/12(20.3)	83(80.6)/18(17.5)	0.831(0.368-1.877)	0.657		
Total bilirubin,mg/dl<br>(≤1/ >1(%); ref=≤1)	51(86.4)/7(11.9)	87(84.5)/15(14.6)	1.256(0.480-3.285)	0.642		
Intraop PRBC<br>(No/Yes(%);ref=No)	45(76.3)/14(23.7)	69(67.0)/34(33.0)	1.584(0.766-3.277)	0.215		
Intraop FFP<br>((No/Yes(%);ref=No)	38(64.4)/21(35.6)	68(66.0)/35(34.0)	0.931(0.476-1.822)	0.835		
Operative time minutes<br>(≤360/ >360(%); ref=≤360)	42(71.2)/17(28.8)	73(70.9)/30(29.1)	1.015(0.501-2.056)	0.966		
Time to surgery,days<br>(≤30/ >30(%); ref=≤30)	16(27.1)/36(61.0)	21(20.4)/73(70.9)	1.545(0.720-3.314)	0.264		
LVI<br>(No/Yes(%);ref=No)	49(83.1)/8(13.6)	70(68.0)/25(24.3)	2.187(0.911-5.252)	0.080	**2.307(0.906-5.874)**	**0.080**
MVI<br>(No/Yes(%);ref=No)	37(62.7)/19(32.2)	66(64.1)/33(32.0)	0.938(0.682-1.290)	0.692		
R1 resection<br>((R0/R2)/R1) (%); ref=R0/R2)	51(86.4)/7(11.9)	94(91.3)/9(8.7)	0.698(0.245-1.983)	0.499		
pT category<br>(T1-2/T3-4(%);ref=T1-T2)	39(66.1)/19(32.2)	81(78.6)/22(21.4)	0.558(0.271-1.149)	0.113		
pN category<br>(N0/N1(%);ref=N0)	35(59.3)/16(27.1)	56(54.4)/41(39.8)	1.602(0.783-3.276)	0.197		
Tumor grading<br>((G1/G2)/(G3/G4) (%);ref= G1/G2)	35(59.3)/19(32.2)	60(58.3)/29(28.2)	0.890(0.436-1.816)	0.750		
ICU time,days	50(84.7)/9(15.3)	77(74.8)/26(25.2)	1.876(0.812-4.334)	0.141		

(≤1/>1(%),ref=1)						
Hospitalization,days (≤14/>14(%); ref=≤14)	35(59.3)/24(40.7)	52(50.5)/51(49.5)	1.430(0.749-2.732)	0.279		
Postoperative complications Clavien-Dindo((0/I/II (%))/(III/IV/V);ref= 0/I/II)	47(79.7)/12(20.3)	81(78.6)/22(21.4)	0.874(0.458-1.669)	0.683		
Liver failure (No/Yes(%);ref=No)	47(79.7)/12(20.3)	87(84.5)/16(15.5)	0.720(0.315-1.649)	0.438		
Bile leak (No/Yes(%);ref=No)	46(78.0)/13(22.0)	87(84.5)/16(15.5)	0.651(0.288-1.469)	0.301		
Hemorrhage (No/Yes(%);ref=No)	55(93.2)/4(6.8)	94(91.3)/9(8.7)	1.316(0.387-4.477)	0.660		
Infection Clavien-Dindo ((0/I/II)/(III/IV/V) (%);ref= 0/I/II)	54(91.5)/5(8.5)	94(91.3)/8(7.8)	0.740(0.362-1.511)	0.408		
Adjuvant therapy (No/Yes(%);ref=No)	39(66.1)/18(30.5)	62(60.2)/38(36.9)	1.328(0.667-2.645)	0.420		
Myosteatosis	No(n=76)	Yes(n=86)	OR (95% CI)	p=	OR (95% CI)	p=
Sex (male/female(%); ref=male)	43(56.6)/33(43.4)	33(38.4)/53(61.6)	2.093(1.117-3.922)	0.021	**3.636(1.447-9.132)**	**0.006**
Age (≤65/ >65 years(%); ref=≤65)	50(65.8)/26(34.2)	30(34.9)/56(65.1)	3.590(1.876-6.870)	< 0.001	**4.989(1.904-13.068)**	**0.001**
ASA ((I/II)/(III/IV) (%);ref= I/II)	30(39.5)/46(60.5)	38(44.2)/48(55.8)	0.824(0.440-1.542)	0.544		
Cholangitis (No/Yes(%);ref=No)	72(94.7)/4(5.3)	80(93.0)/6(7.0)	1.350(0.366-4.976)	0.652		
PVE (No/Yes(%);ref=No)	71(93.4)/5(6.6)	77(89.5)/9(10.5)	1.660(0.531-5.189)	0.384		
Neoadjuvant therapy ((No/Yes(%);ref=No)	67(88.2)/9(11.8)	74(86.0)/12(14.0)	1.207(0.479-3.045)	0.690		
AST, U/L (≤40/ >40(%); ref=≤40)	51(67.1)/25(32.9)	59(68.6)/27(31.4)	0.934(0.482-1.807)	0.838		
ALT, U/L (≤40/ >40(%); ref=≤40)	51(67.1)/25(32.9)	59(68.6)/27(31.4)	0.722(0.351-1.484)	0.375		

Albumin,g/L<br>(≤4.2/ >4.2(%); ref=≤4.2)	22(28.9)/40(52.6)	39(45.3)/28(32.6)	0.395(0.194-804)	0.010	0.817(0.318-2.096)	0.674
AP,U/L<br>(≤100/ >100(%); ref=≤100)	27(35.5)/48(63.2)	31(36.0)/50(58.1)	0.907(0.473-1.739)	0.769		
CRP,mg/l<br>(≤8.2/ >8.2(%); ref=≤8.2)	48(63.2)/25(32.9)	41(47.7)/39(45.3)	1.677(0.882-3.187)	0.115		
GGT,U/L<br>(≤100/ >100(%); ref=≤100)	35(46.1)/40(52.6)	41(47.7)/40(46.5)	0.854(0.455-1.601)	0.622		
Hemoglobin, g/L<br>(≤13/ >13(%); ref=≤13)	25(32.9)/50(65.8)	51(59.3)/34(39.5)	0.333(0.175-0.637)	0.001	0.535(0.230-1.242)	0.145
INR<br>(≤1/>1(%); ref=≤1)	39(51.3)/35(46.1)	44(51.2)/41(47.7)	1.038(0.556-1.938)	0.906		
Platelet count<br>(≤300/ >300(%); ref=≤300)	54(71.1)/21(27.6)	63(73.3)/22(25.6)	0.898(0.446-1.808)	0.763		
Prothrombin time<br>(≤110/ >110(%); ref=≤110)	59(77.6)/15(19.7)	70(81.4)/15(17.4)	0.843(0.381-1.867)	0.673		
Total bilirubin,mg/dl<br>(≤1/ >1(%); ref=≤1)	69(90.8)/6(7.9)	69(80.2)/16(18.6)	2.667(0.985-7.219)	0.054	2.141(0.637-7.196)	0.218
Intraop PRBC<br>(No/Yes(%);ref=No)	62(81.6)/14(18.4)	52(60.5)/34(39.5)	2.896(1.405-5.969)	0.004	1.594(0.594-4.277)	0.354
Intraop FFP<br>((No/Yes(%);ref=No)	54(71.1)/22(28.9)	52(60.5)/34(39.5)	1.605(0.831-3.098)	0.159		
Operative time minutes<br>(≤360/ >360(%); ref=≤360)	55(72.4)/21(27.6)	60(69.8)/26(30.2)	1.135(0.574-2.244)	0.716		
Time to surgery,days<br>(≤30/ >30(%); ref=≤30)	18(23.7)/46(60.5)	19(22.1)/63(73.3)	1.297(0.614-2.742)	0.495		
LVI<br>(No/Yes(%);ref=No)	60(78.9)/10(13.2)	59(68.6)/23(26.7)	2.339(1.025-5.336)	0.043	**2.942(1.047-8.268)**	**0.041**
MVI<br>(No/Yes(%);ref=No)	49(64.5)/23(30.3)	54(62.8)/29(33.7)	0.957(0.819-1.119)	0.585		
R1 resection<br>((R0/R2)/R1) (%); ref=R0/R2)	69(90.8)/6(7.9)	76(88.4)/10(11.6)	1.513(0.523-4.382)	0.445		

pT category (T1-2/T3-4(%);ref=T1-T2)	55(72.4)/20(26.3)	65(75.6)/21(24.4)	0.888(0.437-1.807)	0.744		
pN category (N0/N1(%);ref=N0)	47(61.8)/25(32.9)	44(51.2)/32(37.2)	1.367(0.703-2.660)	0.357		
Tumor grading ((G1/G2)/(G3/G4) (%);ref= G1/G2)	45(59.2)/22(28.9)	50(58.1)/26(30.2)	1.064(0.530-2.134)	0.862		
ICU time,days (≤1/>1(%),ref=1)	62(81.6)/14(18.4)	65(75.6)/21(24.4)	1.431(0.669-3.061)	0.356		
Hospitalization,days (≤14/>14(%); ref=≤14)	45(59.2)/59(56.2)	38(38.4)/61(61.6)	1.521(0.816-2.835)	0.187		
Postoperative complications Clavien-Dindo ((0/I/II)/(III/IV/V) (%);ref= 0/I/II)	47(61.8)/29(38.2)	47(54.7)/39(45.3)	1.345(0.718-2.520)	0.355		
Liver failure (No/Yes(%);ref=No)	66(86.8)/10(13.2)	68(79.1)/18(20.9)	1.747(0.751-4.063)	0.195		
Bile leak (No/Yes(%);ref=No)	62(81.6)/14(18.4)	71(82.6)/15(17.4)	0.936(0.419-2.091)	0.871		
Hemorrhage (No/Yes(%);ref=No)	72(94.7)/4(5.3)	77(89.5)/9(10.5)	2.104(0.621-7.132)	0.232		
Infection Clavien-Dindo ((0/I/II)/(III/IV/V) (%);ref= 0/I/II)	72(94.7)/3(3.9)	76(88.4)/10(11.6)	1.004(0.499-2.022)	0.991		
Adjuvant therapy (No/Yes(%);ref=No)	39(51.3)/33(43.4)	62(72.1)/23(26.7)	0.438(0.225-0.854)	0.015	0.485(0.205-1.146)	0.099
VFA(cm²)	< 100(n=61)	≥100(n=101)	OR (95% CI)	p=	OR (95% CI)	p=
Sex (male/female(%); ref=male)	13(21.3)/48(78.7)	63(62.4)/38(37.6)	0.163(0.078-0.340)	< 0.001	**0.247(0.114-0.536)**	**< 0.001**
Age (≤65/ >65 years(%); ref=≤65)	39(63.9)/22(36.1)	41(40.6)/60(59.4)	2.594(1.346-5.001)	0.004	**3.577(1.592-8.035)**	**0.002**
ASA ((I/II)/(III/IV) (%);ref= I/II)	27(44.3)/34(55.7)	41(40.6)/60(59.4)	1.162(0.611-2.210)	0.647		
Cholangitis (No/Yes(%);ref=No)	56(91.8)/5(8.2)	96(95.0)/5(5.0)	0.583(0.162-2.104)	0.410		

PVE (No/Yes(%);ref=No)	57(93.4)/4(6.6)	91(90.1)/10(9.9)	1.566(0.469-5.230)	0.466		
Neoadjuvant therapy ((No/Yes(%);ref=No)	54(88.5)/7(11.5)	87(86.1)/14(13.9)	1.241(0.471-3.271)	0.662		
AST, U/L (≤40/ >40(%); ref=≤40)	39(63.9)/22(36.1)	71(70.3)/30(29.7)	0.749(0.381-1.471)	0.401		
ALT, U/L (≤40/ >40(%); ref=≤40)	35(57.4)/16(26.2)	62(61.4)/27(26.7)	0.953(0.453-2.005)	0.898		
Albumin,g/L (≤4.2/ >4.2(%); ref=≤4.2)	24(39.3)/25(41.0)	37(36.3)/43(42.2)	1.116(0.547-2.274)	0.763		
AP,U/L (≤100/ >100(%); ref=≤100)	17(27.9)/40(65.6)	41(40.6)/58(57.4)	0.601(0.300-1.204)	0.151		
CRP,mg/l (≤8.2/ >8.2(%); ref=≤10)	30(49.2)/26(42.6)	52(51.5)/45(44.6)	0.999(0.516-1.931)	0.996		
GGT,U/L(≤100/ >100(%); ref=≤100)	28(45.9)/29(47.5)	48(47.5)/51(50.5)	1.026(0.535-1.969)	0.939		
Hemoglobin, g/L (≤13/ >13(%); ref=≤13)	37(60.7)/22(36.1)	39(38.6)/62(61.4)	2.674 (1.378-5.186)	0.004	**3.265(1.455-7.325)**	**0.004**
INR (≤1/>1(%); ref=≤1)	36(59.0)/23(37.7)	47(46.5)/53(52.5)	1.765(0.918-3.395)	0.089	1.310(0.603-2.844)	0.495
Platelet count (≤300/ >300(%); ref=≤300)	40(65.6)/19(31.1)	77(76.2)/24(23.8)	0.656(0.322-1.338)	0.247		
Prothrombin time (≤110/ >110(%); ref=≤110)	44(72.1)/15(24.6)	85(84.2)/15(14.9)	0.518(0.232-1.155)	0.108		
Total bilirubin,mg/dl (≤1/ >1(%); ref=≤1)	49(80.3)/10(16.4)	89(88.1)/12(11.9)	0.661(0.266-1.639)	0.371		
Intraop PRBC (No/Yes(%);ref=No)	39(63.9)/22(36.1)	75(74.3)/26(25.7)	0.615(0.309-1.222)	0.165		
Intraop FFP ((No/Yes(%);ref=No)	39(63.9)/22(36.1)	67(66.3)/34(33.7)	0.900(0.462-1.751)	0.755		
Operative time minutes (≤360/ >360(%); ref=≤360)	44(72.1)/17(27.9)	71(70.3)/30(29.7)	1.094(0.541-2.211)	0.803		

Time to surgery,days (≤30/ >30(%); ref=≤30)	17(27.9)/38(62.3)	20(19.8)/71(70.3)	1.588(0.745-3.386)	0.231		
LVI (No/Yes(%);ref=No)	45(73.8)/11(18.0)	74(73.3)/22(21.8)	1.216(0.539-2.742)	0.637		
MVI (No/Yes(%);ref=No)	34(55.7)/24(39.3)	69(68.3)/28(27.7)	0.575(0.290-1.138)	0.112		
R1 resection ((R0/Rx)/R1) (%); ref=R0/Rx)	46(75.4)/15(24.6)	87(86.1)/13(12.9)	0.458(0.201-1.045)	0.063		
pT category (T1-2/T3-4(%);ref=T1-T2)	45(73.8)/15(24.6)	75(74.3)/26(25.7)	1.040(0.499-2.169)	0.917		
pN category (N0/N1(%);ref=N0)	37(60.7)/18(29.5)	54(53.5)/39(38.6)	1.485(0.739-2.983)	0.267		
Tumor grading ((G1/G2)/(G3/G4) (%);ref= G1/G2)	40(65.6)/14(23.0)	55(54.5)/34(33.7)	1.766(0.839-3.716)	0.134		
ICU time,days (≤1/>1(%),ref=1)	49(80.3)/12(19.7)	78(77.2)/23(22.8)	1.204(0.550-2.637)	0.643		
Hospitalization,days (≤14/>14(%); ref=≤14)	26(42.6)/35(57.4)	61(60.4)/40(39.6)	0.487(0.255-0.929)	0.029	0.570(0.260-1.251)	0.161
Postoperative complications Clavien-Dindo ((0/I/II)/(III/IV/V) (%);ref= 0/I/II)	45(73.8)/16(26.2)	63(62.4)/38(37.6)	0.614(0.323-1.167)	0.136		
Liver failure (No/Yes(%);ref=No)	47(77.0)/14(23.0)	87(86.1)/14(13.9)	0.540(0.238-1.228)	0.142		
Bile leak (No/Yes(%);ref=No)	46(75.4)/15(24.6)	87(86.1)/14(13.9)	0.493(0.219-1.111)	0.088	0.636(0.229-1.765)	0.385
Hemorrhage (No/Yes(%);ref=No)	55(90.2)/6(9.8)	94(93.1)/7(6.9)	0.683(0.218-2.135)	0.512		
Infection Clavien-Dindo ((0/I/II)/(III/IV/V) (%);ref= 0/I/II)	42(68.9)/18(29.5)	76(75.2)/25(24.8)	0.758(0.371-1.546)	0.445		
Adjuvant therapy (No/Yes(%);ref=No)	39(63.9)/21(34.4)	63(61.8)/35(34.3)	1.048(0.535-2.055)	0.891		
Sarcoprnic_obesity	No(n=137)	Yes(n=25)	OR (95% CI)	p=	OR (95% CI)	p=

Sex<br>(male/female(%); ref=male)	55(40.1)/82(59.9)	21(84.0)/4(16.0)	0.128(0.042-0.393)	< 0.001	**0.180(0.056-0.582)**	**0.004**
Age<br>(≤65/ >65 years(%); ref=≤65)	74(54.0)/63(46.0)	6(24.0)/19(76.0)	3.720(1.400-9.885)	0.008	**4.007(1.375-11.674)**	**0.011**
ASA<br>((I/II)/(III/IV) (%);ref= I/II)	60(43.8)/77(56.2)	8(32.0)/17(68.0)	1.656(0.669-4.095)	0.275		
Cholangitis<br>(No/Yes(%);ref=No)	127(92.7)/10(7.3)	0(0)/25(100.0)	0(0-0)	0.999		
PVE<br>(No/Yes(%);ref=No)	125(91.2)/12(8.8)	23(92.0)/2(8.0)	0.906(0.190-4.317)	0.901		
Neoadjuvant therapy<br>((No/Yes(%);ref=No)	120(87.6)/17(12.4)	21(84.0)/4(16.0)	1.345(0.412-4.392)	0.624		
AST, U/L<br>(≤40/ >40(%); ref=≤40)	90(65.7)/47(34.3)	20(80.0)/5(20.0)	0.479(0.169-1.356)	0.166		
ALT, U/L<br>(≤40/ >40(%); ref=≤40)	82(59.9)/37(27.0)	15(60.0)/6(24.0)	0.886(0.319-2.466)	0.817		
Albumin,g/L<br>(≤4.2/ >4.2(%); ref=≤4.2)	53(38.7)/53(38.7)	8(32.0)/15(60.0)	1.875(0.733-4.794)	0.189		
AP,U/L<br>(≤100/ >100(%); ref=≤100)	45(32.8)/86(62.8)	13(52.0)/12(48.0)	0.483(0.204-1.145)	0.099	1.009(0.292-3.479)	0.989
CRP,mg/l<br>(≤8.2/ >8.2(%); ref=≤8.2)	71(51.8)/57(41.6)	18(72.0)/7(28.0)	0.599(0.247-1.454)	0.257		
GGT,U/L<br>(≤100/ >100(%); ref=≤100)	59(43.1)/72(52.6)	17(68.0)/8(32.0)	0.386(0.156-0.956)	0.040	0.405(0.134-1.225)	0.110
Hemoglobin, g/L<br>(≤13/ >13(%); ref=≤13)	67(48.9)/68(49.6)	9(36.0)/14(64.0)	1.752(0.724-4.238)	0.214		
INR<br>(≤1/>1(%); ref=≤1)	75(54.7)/60(43.8)	8(32.0)/16(64.0)	2.500(1.002-6.236)	0.049	1.247(0.419-3.714)	0.691
Platelet count<br>(≤300/ >300(%); ref=≤300)	95(69.3)/40(29.2)	22(88.0)/3(12.0)	0.324(0.092-1.144)	0.080	0.779(0.184-3.299)	0.735
Prothrombin time<br>(≤110/ >110(%); ref=≤110)	107(78.1)/28(20.4)	22(88.0)/2(8.0)	0.347(0.077-1.567)	0.169		

Total bilirubin,mg/dl (≤1/ >1(%); ref=≤1)	116(84.7)/19(13.9)	22(88.0)/3(12.0)	0.833(0.229-3.055)	0.782		
Intraop PRBC (No/Yes(%);ref=No)	95(69.3)/42(30.7)	19(76.0)/6(24.0)	0.714(0.266-1.917)	0.504		
Intraop FFP ((No/Yes(%);ref=No)	88(64.2)/49(35.8)	18(72.0)/7(28.0)	0.698(0.273-1.789)	0.454		
Operative time minutes (≤360/ >360(%); ref=≤360)	94(68.6)/43(31.4)	21(84.0)/4(16.0)	0.416(0.135-1.287)	0.128		
Time to surgery,days (≤30/ >30(%); ref=≤30)	33(24.1)/90(65.7)	4(16.0)/19(76.0)	1.742(0.552-5.498)	0.344		
LVI (No/Yes(%);ref=No)	103(75.2)/25(18.2)	16(64.0)/8(32.0)	2.060(0.793-5.351)	0.138		
MVI (No/Yes(%);ref=No)	82(59.9)/48(35.0)	21(84.0)/4(16.0)	0.325(0.105-1.004)	0.051	0.438(0.128-1.498)	0.188
R1 resection ((R0/R2)/R1) (%); ref=R0/R2)	121(88.3)/15(10.9)	21(96.0)/1(4.0)	0.336(0.042-2.666)	0.302		
pT category (T1-2/T3-4(%);ref=T1-T2)	99(72.3)/37(27.0)	21(84.0)/4(16.0)	0.510(0.164-1.584)	0.244		
pN category (N0/N1(%);ref=N0)	75(54.7)/51(37.2)	16(64.0)/6(24.0)	0.551(0.202-1.504)	0.245		
Tumor grading ((G1/G2)/(G3/G4) (%);ref= G1/G2)	79(57.7)/43(31.4)	16(64.0)/5(20.0)	0.574(0.197-1.675)	0.310		
ICU time,days (≤1/>1(%),ref=1)	108(78.8)/29(21.2)	19(76.0)/6(24.0)	1.176(0.430-3.214)	0.752		
Hospitalization,days (≤14/>14(%); ref=≤14)	69(50.4)/68(49.6)	18(72.0)/7(28.0)	0.395(0.155-1.005)	0.051	2.382(0.453-12.525)	0.306
Postoperative complications Clavien-Dindo ((0/I/II)/(III/IV/V) (%);ref= 0/I/II)	74(54.0)/63(46.0)	20(80.0)/5(20.0)	0.294(0.104-0.827)	0.020	**0.246(0.080-0.760)**	**0.015**
Liver failure (No/Yes(%);ref=No)	111(81.0)/26(19.0)	23(92.0)/2(8.0)	0.371(0.082-1.675)	0.197		
Bile leak	109(79.6)/28(20.4)	24(96.0)/1(4.0)	0.162(0.021-1.251)	0.081	0.363(0.036-3.718)	0.394

(No/Yes(%);ref=No)						
Hemorrhage (No/Yes(%);ref=No)	124(90.5)/13(9.5)	130(81.3)/30(18.8)	0(0-0)	0.999		
Infection Clavien-Dindo ((0/I/II)/(III/IV/V) (%);ref= 0/I/II)	96(70.1)/40(29.2)	22(88.0)/3(12.0)	0.327(0.093-1.155)	0.083	0.894(0.096-8.339)	0.921
Adjuvant therapy (No/Yes(%);ref=No)	83(60.6)/49(35.8)	18(72.0)/7(28.0)	0.659(0.257-1.689)	0.385		

Multiple Variables were associated with altered body composition in intrahepatic cholangiocarcinoma. Variables displaying a p value < 0.05 in the univariate analysis were transferred into a multivariable Logistic regression model.

Abbreviations: ALT, alanine aminotransferase; AP, Alkaline phosphatase; ASA, American Society of Anesthesiologists; AST, aspartate aminotransferase; BMI, body mass index; CRP, C--reactive protein; F, female; FFP, Fresh frozen plasma; GGT, gamma-glutamyl transferase; HR, hazard ratio; iCCA, intrahepatic cholangiocarcinoma; ICU, intensive care unit; INR, international normalized ratio; LVI, lymph vascular invasion; M, male; MVI, microvascular invasion; PRBC, Packed Red Blood Cells;PVE, portal vein embolization; RFS, Recurrence free survival.
